# Correlations between Endothelial Functions and ROS Detection in Diabetic Microvascular Wall: *Early and Late Ascorbic Acid Supplementation*

**DOI:** 10.1155/2012/709695

**Published:** 2012-05-28

**Authors:** Pattarin Sridulyakul, Natchaya Wongeak-in, Suthiluk Patumraj

**Affiliations:** ^1^Department of Biology, Faculty of Science, Srinakharinwirot University, Bangkok 10110, Thailand; ^2^Center of Excellence for Microcirculation, Department of Physiology, Faculty of Medicine, Chulalongkorn University, Bangkok 10330, Thailand

## Abstract

The correlation between endothelial function and reactive oxygen species detecting from diabetic microvascular wall and the antioxidant effect of ascorbic acid (AA) during early and late phases of diabetic induction were determined. Male Spraque-Dawley rats were divided into four groups: control, diabetes rats (DM, using iv.injection of 55 mg/kg BW streptozotocin, (STZ)), and two groups of DM rats treated with AA (1 g/L, (STZ)) starting on day 2 (DM + AAday2) and week 6th (DM + AA6wk). On 12th week after STZ injection, the findings showed that in DM group, Ach (10^−5^ M)-induced vasodilatation was decreased, while the number of leukocyte adhesion was increased significantly (*P* < 0.01). Interestingly, these abnormalities induced by DM could be protected or improved in both AA-treated groups, DM + AAday2 and DM + AA6wk. By using dihydrorhodamine 123, our findings also indicated that the existing of ROS productions on diabetic arteriolar and venular walls were different significantly (ROS_arteriole_ = 165.89 ± 24.59 and ROS_venule_ = 172.26 ± 34.70) (*P* < 0.05). Moreover by using BH4 inhibitor to induce increase in arteriolar ROS, the results also confirmed that AA could improve endothelial function with closed correlation to its potential to reduce vascular ROS content.

## 1. Introduction

 The imbalance between hyperglycemic-induced reactive oxygen species (ROS) and antioxidative systems both enzymatic and nonenzymatic appears to be a major factor contributed to several diabetic complications [1–6]. In particular, it has been suggested that the diabetes-induced oxidative stress is a crucial factor contributing to endothelial cell dysfunction. The potential of endothelial cell line both arteriolar and venular vessels to produce NO depending on flow-mediated or other agonists is physiological significance. Many studies have shown close correlation between diabetes-induced ROS and impairments of both endothelial functions at arterioles and venules in the microcirculation. In both human and animal diabetic models, two characters which are commonly used as indicators of endothelial cell dysfunction are the impairment of endothelium-dependent vascular relaxation and the increase in leukocyte-endothelial cell interaction [4–9]. Particularly, the decrease of nitric oxide (NO) bioavailability is referred as the underlining cause of those impairments [5, 9].

Interestingly, the supplementations of antioxidants, such as vitamin C and vitamin E, have been used for protecting endothelium against ROS damages [5, 10, 11]. However, there was no evidence demonstrated whether the supplementation of ascorbic acid (AA) that could restore the diabetes-induced endothelial dysfunction is associated directly with its action particularly on diabetes-induced ROS at the vascular wall of arterioles and venules. Therefore, the present study was aimed to examine the correlation between changes of endothelial function and microvascular wall ROS content. The direct detection of microvascular wall ROS content will be accessed by dihydrorhodamine 123 (DHR) in the mesenteric arteriolar and venular walls of diabetes and diabetes treated with AA groups.

In order to confirm the beneficial effect of ascorbic acid on protecting endothelial function, the acute change in Ach-response after pretreated with BH_4_-inhibitor, 2,4-diamino-6-hydroxy-pyrimidine (DAHP, Sigma-Aldrich Co., USA), was further conducted in normal rat group. This experimental DAHP-induced BH_4_ deficiency was set up in order to imitate the condition of diabetes-induced endothelial dysfunction. In addition, this kind of research findings could also lead to the possible mechanism of how vitamin C could protect diabetic endothelium.

## 2. Materials and Methods

### 2.1. Diabetic Induction

Male Spraque-Dawley rats (200–250 g) were purchased from the National Laboratory Animal Center, Salaya Campus, Thailand. Experiments were conducted in accordance with the guidelines for experimentation with animals of the National Research Council of Thailand (1999) and approved by Ethical Committee, Faculty of Pharmacy, Chulalongkorn University. Diabetes was induced by intravenous injection of streptozotocin (STZ; 50 mg/kg·BW) (Sigma Chemical Co., USA). The inclusion criteria for diabetic condition were the level of blood glucose ≥200 mg/dL at 48 hours after the STZ injection. Control rats were injected by the same volume of citrate buffer instead.

### 2.2. Experimental Protocol

The animals were divided into four groups: control (CON), diabetic (DM), diabetic rats treated with ascorbic acid (AA) starting on day 2 (DM + AAday2) and on 6 weeks (DM + AA6wk) after STZ injection. In DM + AAday2, and DM + AA6wk groups, AA (99% L-ascorbic acid, Sigma Chemical Co., USA) was started to administer in drinking water (1 g/L) on 2 days and 6 weeks after STZ injections, respectively.

### 2.3. Microscopic Observation of the Mesenteric Microcirculation

 The rats were anesthetized with sodium pentobarbital (50 mg/kg·BW, iv). The mesenteric microvasculature was observed using fluorescence videomicroscopy. A chamber was placed under the distal ileum mesentery, which was superfused continuously with Krebs–Ringer buffer solution (pH 7.4,2 mL/min, 37°C) and covered with buffer-soaked gauze to prevent tissue dehydration. Mesenteric microcirculation was observed by fluorescence videomicroscopic system (Optiphot 2, Nikon, Japan) equipped with a 100 W mercury lamp, real-time CCD camera (C2400, Hamamatsu Photonics, Japan), a video recorder (VC-S5, Sharp, Japan) with a video timer (VTG-33, For-A, Japan), and a 20x objective lens (CF Plan Fluor, Nikon, Japan). Fluorescein isothiocyanate labeled dextran (FITC-Dx-250; MW 250,000, Sigma-Aldrich Co, USA) (5 *μ*g/mL) and acrdine orange (Sigma-Aldrich Co, USA) (25 *μ*g/mL) were injected intravenously for visualizing the mesenteric microvasculature and the behavior of leukocytes in microvessels, respectively [4, 7].

### 2.4. *In Vivo* Assessment of ROS Using DHR

To quantify the amount of ROS located at the vascular walls of selected mesenteric area, the superfusion of 0.1 mL/min dihydrorhodamine 123 (DHR, Sigma Chemical Co., USA) (10 *μ*mole/L) was used and recorded by intravital fluorescence videomicroscopy. DHR, an oxidant-sensitive fluorescent probe, was used to measure ROS generation *in vivo*. This method has been previously validated by different authors [12–15]. Oxidation of DHR primarily by hydrogen peroxide-dependent reactions forms rhodamine 123, which fluoresces. The amount of formed rhodamine 123 was followed by excited wavelength, 488 nm, and emission wavelength, 525 nm, respectively [12–14]. Images of 512 × 512 pixels were acquired by 20x objective lens. Using a digital image software (Image Pro Plus; Media Cybernetics, Inc, USA), the rhodamine 123 fluorescence intensity of each small window located along the 100-**μ**m length of arterioles (15–30 *μ*m) and venules (20–30 *μ*m) were determined. The averages of intensities of 14 windows (100-**μ**m length) was determined for background intensity (0 min before DHR superfusion, *I*_base_) and for 1-min intensity (*I*_1min⁡_) after DHR superfusion. The percentage of ROS change was then presented by the following:

(1) $$\begin{array}{cc} & \%\;\text{change}\;\text{of}\;\text{ROS}-\text{associated}\;\text{fluorescent}\;\text{intensity}\\ & \;\;=\left(I_{1\min}-\frac{I_{\text{base}}}{I_{\text{base}}}\right)\times 100. \end{array}$$

### 2.5. Measurement of Biochemical and Physiological Parameters

 The arterial blood pressure was measured via a canula inserted into the carotid artery using a pressure transducer (Nikhon Koden, Japan). Blood glucose, plasma AA, and glycosylated hemoglobin (HbA_1C_) were determined in blood sample collected at the end of each experiment. Blood glucose was determined using a glucometer (Advance Glucometer, Boehringer Mannheim, Germany). The HbA_1C_ was analyzed using colorimetry (Bangkok RIA lab Co, Thailand). The plasma AA was measured using enzyme-assisted spectrophotometry (Research Center, Ramathibodi Hospital, Mahidol University, Thailand).

### 2.6. Measurement of Vascular Function

 Two different vasodilators, acetylcholine (Ach; 10^−5^ M) and sodium nitroprusside (SNP; 10^−5^ M) were used to examine the endothelial-dependent and -independent vasodilatation responses of mesenteric arterioles (15 to 30 *μ*m in diameter). Changes of arteriolar diameters were determined by off-line image analysis (Image Pro Plus; Media Cybernetics, USA), based on the fluorescence images recorded 5 minutes after the topical application of each vasodilator and expressed as the percentage changes (%) from the baseline values (NE-pre-constricted (NE; 10^−5^ M)).

### 2.7. Measurement of Number of Adherent Leukocytes

 In order to assess the number of leukocyte adherence, the mesenteric tissues were prepared similarly to that described earlier. Instead of using FITC-dextran 250, 0.3 mL rhodamine 6G (conc. 0.3 mg/mL; Sigma, St. Louis, USA), a total of 0.09 mg rhodamine per animal, was injected into the rat's jugular vein [9]. Based on the rhodamine video images of each experiment, we counted the number of leukocytes (*N*) which adhered to mesenteric venules (20–30 *μ*m in diameter) and remained stationary for more than 30 seconds. The number of leukocyte adhering was counted by using the software Global lab image II. Cn, *cells/100 *μ*m length* of *venule*, was determined by the following:

(2) $$\text{Cn}=\frac{N}{L}\times 100,$$

where *N* is the total number of adherent leukocytes and *L* is the venular length (**μ**m) under the measurement.

### 2.8. To Confirm the Antioxidative Effect of Ascorbic Acid on Endothelial Function

This study was performed by using 2,4-diamino-6-hydroxypyrimidine (20 mM; DAHP, Sigma-Aldrich Co., USA), 6R-5,6,7,8-tetrahydro-L-biopterin dihydrochloride (0.1 mM; 6R-BH4, Sigma-Aldrich Co., USA), and 2.6 mM L-ascorbic acid (Sigma-Aldrich Co., USA). DAHP is a compound that reduces intracellular BH_4_ levels. It is a selective, specific inhibitor of GTP-CH 1, a rate limiting enzyme for de novo BH_4_ synthesis [16–18].

In our experiment, we used 6R-BH_4_ as a positive control, which is a compound for increased intracellular BH_4_ [19]. The 6R-BH_4_ is a cofactor for eNOS and binds to the enzyme at a ratio of 1 : 1 BH_4_ : eNOS [20].

The rats were separated into four groups: control (CON + vehicle), control treated with DAHP (CON + DAHP), control treated with DAHP with ascorbic acid (CON + DAHP + AA), and control treated with DAHP with ascorbic acid and BH_4_ (CON + DAHP + AA + BH_4_).

**Figure 1:**
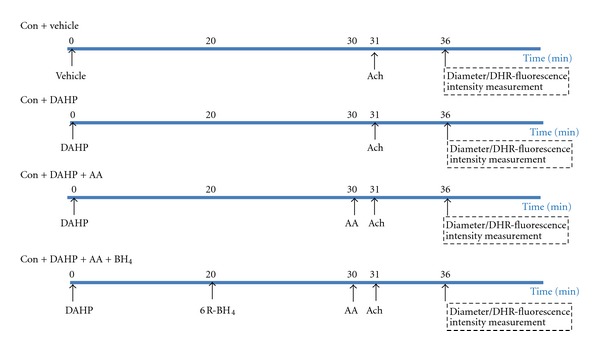
Experimental protocol for measuring ROS content and vascular response to Ach (acetylcholine) as the effect of AA (ascorbic acid) and/or 6R-BH_4_ (6R-5,6,7,8-tetrahydro-L-biopterin dihydrochloride) application after inhibiting BH_4_ biosynthetic by DAHP (2,4-diamino-6-hydroxypyrimidine). There are four groups: control (CON + vehicle), control treated with DAHP (CON + DAHP), control treated with DAHP with ascorbic acid (CON + DAHP + AA), and control treated with DAHP with ascorbic acid and BH_4_ (CON + DAHP + AA + BH_4_).

 To inhibit intracellular BH_4_ levels, we applied DAHP (20 mM; 1 mL/5 min for 30 min), in order to diminish NO production. In the presence of DAHP, vascular response to Ach (10 *μ*M; 1 mL/5 min) was obtained after application of AA (2.6 mM; 10 mL/1 min) plus 6R-BH_4_ (0.1 mM; 1 mL/5 min for 10 min). Moreover, the arterial ROS content was also detected using DHR-fluorescent probe (dihydrorhodamine 123 Sigma Chemical Co., USA) (10 *μ*mole/L) as well. The experimental protocol of each group was shown in Figure 1.

### 2.9. Statistical Analyses

All data were expressed as means ± SE. One-way analysis of variance with Tukey's Post Hoc test was used to compare between mean values. Significant difference was considered by a probability (*P-*value) less than or equal to 0.05.

## 3. Results

### 3.1. Biochemical Parameters and Physiological Characteristics

The intravenous injection of STZ (50 mg/kg·BW) caused pancreatic islet cells to damage, resulting in hyperglycemia within 48 hours. The hyperglycemia maintained throughout the 12-weeks period of experiment. Loss of body weight was observed in all of three DM groups, DM, DM + AAday2, and DM + AA6wk, as compared to their aged-match controls. In both DM + AAday2, and DM + AA6wk groups, the blood glucose and HbA_1C_ were not significantly different when compared to the DM rats, while AA supplementation increased the body weight significantly (*P* < 0.001) (Table 1). Plasma AA level was reduced significantly in DM group as compared to the controls. However, this reduction did not appear in both DM + AAday2 and DM + AA6wk groups (Table 1). The mean arterial blood pressure (MAP) was increased in DM group. The increase in MAP was attenuated significantly with AA supplementation in both DM + AAday2 and DM + AA6wk groups (*P* < 0.001) (Table 1).

### 3.2. Effect of Ascorbic Acid on Vascular Function

**Figure 2:**
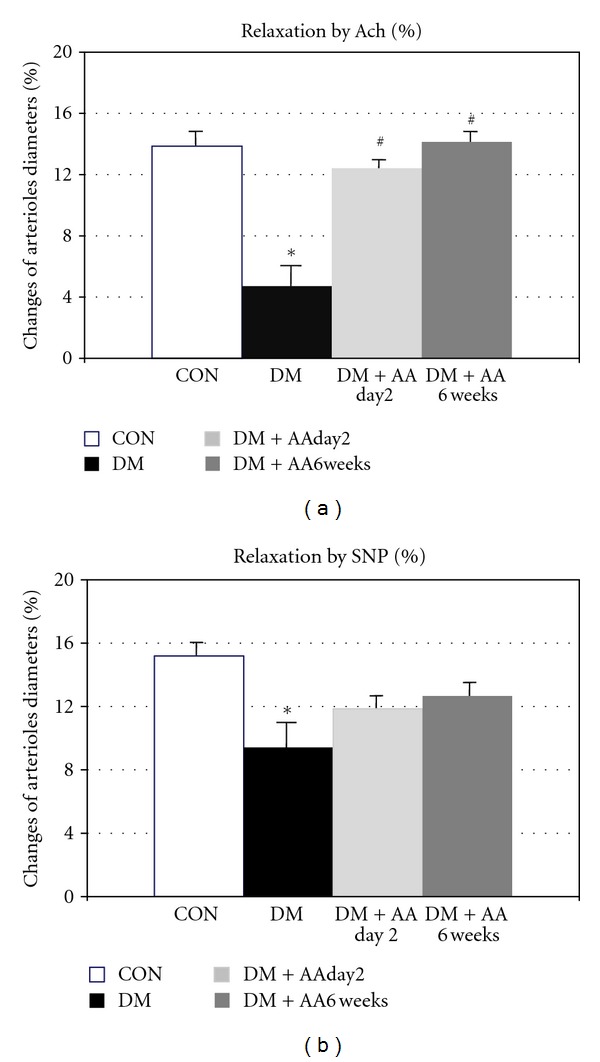
Percentage relaxation to Ach 10^−5^ M and SNP 10^−5^ M in control (CON), diabetes (DM), diabetes treated with vitamin C (1g/L) starting on day 2 (DM + AAday2) and on 6 weeks (DM + AA6wk) after STZ injection. Values are mean ± SEM, CON: *n* = 9, DM: *n* = 7, DM + AAday2: *n* = 7, DM + AA6wk: *n* = 6. *P < 0.001 versus CON, ^#^ *P* < 0.01 versus DM.

Figures 2(a) and 2(b) showed the percentage changes of arteriolar diameters after relaxing by Ach and SNP in control, diabetes rats without and with AA supplementation. It is to be noted that AA supplementation prevented and restored the endothelium-dependent vasodilatation, but not for the SNP vasodilatation.

### 3.3. Effect of Ascorbic Acid on Leukocytes-Endothelial Cell Interaction

**Figure 3:**
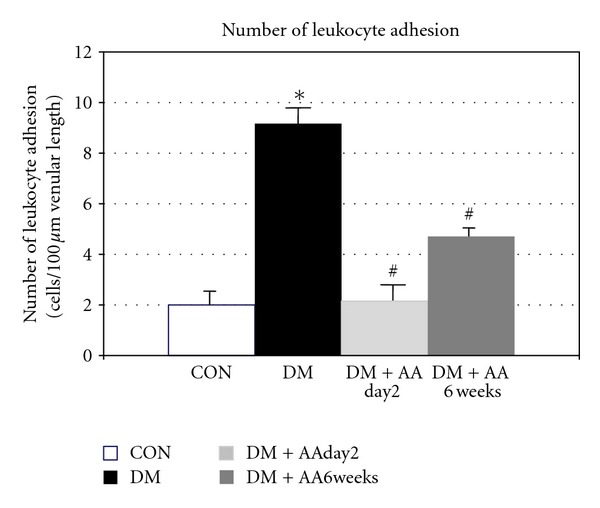
Number (Cn) of leukocyte adhesion in control (CON), diabetes (DM), diabetes treated with vitamin C (1g/L) starting on day 2 (DM + AAday2) and on 6 weeks (DM + AA6wk) after STZ injection. Values are mean ± SEM, CON (*n* = 8), DM (*n* = 6), DM + AAday2 (*n* = 7), and DM + AA6wk (*n* = 7). *P < 0.001 versus CON, ^#^ *P* < 0.01 versus DM.

Figure 3 showed the number of leukocyte adhesion in CON, DM, DM + AAday2 and DM + AA6wk groups. Their adherent-leukocyte densities were changed from 9.17 ± 1.45 cells/100 *μ*m in DM group to 4.71 ± 0.43 cells/100 *μ*m in DM + AAday2 group and to 2.15 ± 0.31 cells/100 *μ*m in DM + AA6wk group, respectively. Interestingly, the number of leukocyte adhesion was decreased significantly by both intervals of AA supplementation.

### 3.4. Effect of Ascorbic Acid on Diabetes-Induced ROS Production

**Figure 4:**
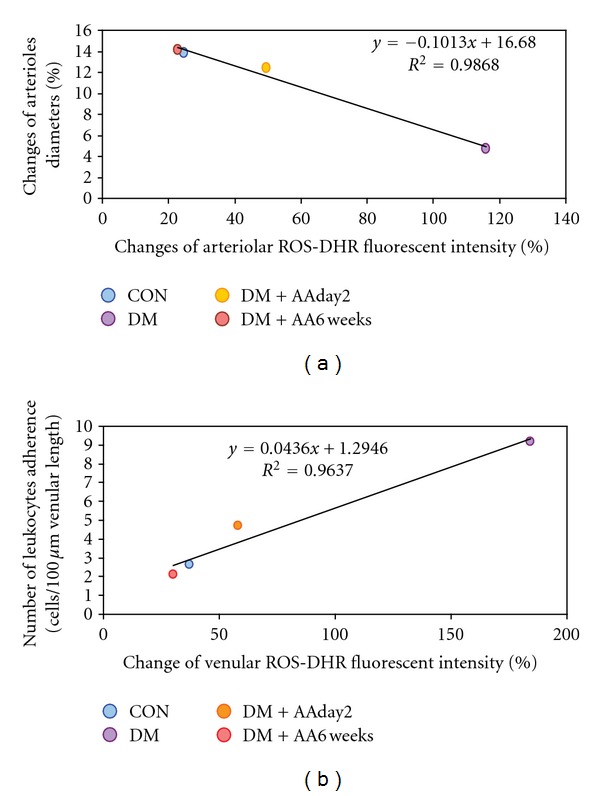
Percentage of changed DHR intensities and number (Cn) of leukocyte-endothelial cell interaction in each group of control (CON), diabetes (DM), diabetes treated with vitamin C (1 g/L) starting on day 2 (DM + AAday2), and on 6 weeks (DM + AA6wk) after STZ injection was plotted. The correlation and regression line were obtained for each group as demonstrated in (a). In (b), the correlation and regression line were obtained for combined data of every group.

Figure 4 showed the changes in the ROS-dependent fluorescence intensities* detected *from the rat mesenteric arterioles and venules of each group at 0 and 1 minute after DHR-123 superfusion. It was observed that DM showed the strongly increased fluorescence intensity as early as 1 min after DHR-123 superfusion. The changes in fluorescence intensities from CON, DM, DM + AAday2, and DM + AA6wk groups after 1-min DHR superfusion were analyzed using digital image analysis as described in methodology above. The averages ROS contents determined from both vascular walls of each group (CON, DM, DM + AAday2, and DM + AA6wk) were summarized in Table 2. These findings indicated that early AA supplementation as well as delayed supplementation of AA could similarly attenuate the increase in ROS generation at both sites of microvascular walls. Interestingly, our findings also indicated that the existing of ROS productions on diabetic arteriolar and venular walls (Table 2) were ROS_arteriole_ = 165.89 ± 24.59 and ROS_venule_ = 172.26 ± 34.70. (*P* < 0.05).

### 3.5. Correlations between Arteriolar ROS Generation versus Vasodilatation and Venular ROS Generation versus Leukocytes Adhesion

Figures 4(a) and 4(b) are plotted between arteriolar and venular ROS productions against Ach-vasodilatation (results in Figure 2(a)) and number of leukocyte adhesion (results in Figure 3), respectively. By using Pearson correlation and linear regression analysis, the relationships between those two parameters of each group were determined as follows:

Arteriolar wall:

(3) $$y_{a}=-0.101x_{a}+16.68\;\left(R^{2}\;=\;0.99,P<0.02\right)$$

Venular wall:

(4) $$y_{v}=0.044x_{v}+1.29\;\left(R^{2}=0.96,\;P<0.018\right),$$

where *y*_*a*_ and *y*_*v*_ are % change of arteriolar diameter in response to Ach, and number of leukocyte adhesion, respectively. *x*_*a*_ and *x*_*v*_ are % change of ROS contents in arteriolar and venular walls, respectively.

### 3.6. Testing the Effect of Ascorbic Acid on Protecting Endothelial Function Using DAHP and the BH_4_-Inhibitor

**Figure 5:**
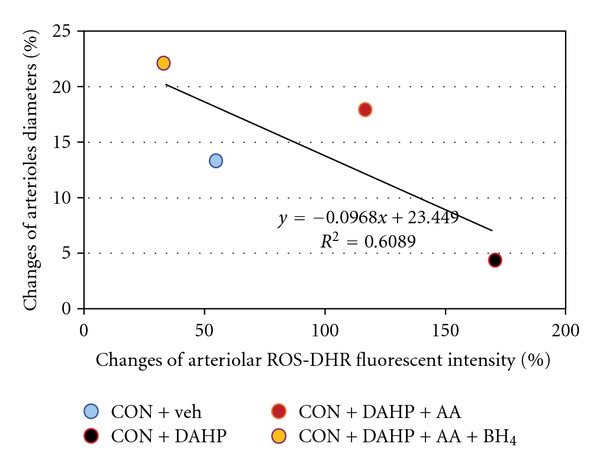
The correlation and regression line between percentage changes of ROS (intensities/100-*μ*m arteriolar length) and arteriolar diameters in response to Ach from mesenteric arterioles in the presence or absence of BH_4_ synthetic inhibitor were obtained when combined the data of every group.

The results showed in Table 3 indicated that the ROS-dependent fluorescence intensity was significantly increased when BH_4_ synthesis was blocked by DAHP in relation to CON + Veh group (DAHP-treated CON = 169.61 ± 6.46% and CON + Veh = 54.48 ± 14.90%, resp.). Whereas the enhanced ROS content was significantly decreased in AA and 6R-BH_4_ treated groups (CON + DAHP + AA + BH_4_ = 33.89 ± 7.62%, resp.). Moreover, AA in combination with BH_4_-treated group showed synergistic increase in Ach-induced vasodilation when compared to CON + DAHP. By using Pearson correlation and linear regression analysis, the relationship between ROS-dependent fluorescence intensity and Ach-response can be described by the linear equation: *y* = −0.097*x* + 23.45; *R* ^2^ = 0.61, as shown in Figure 5.

## 4. Discussion

The results showed in Tables 1 and 2 demonstrated that diabetes promoted the elevations of blood glucose, HbA_1C_, and mean arterial blood pressure. Whereas, the depletion of plasma vitamin C was showed with the increased vascular ROS-sensitive DHR-123 intensities. The results of ROS-sensitive fluorescent probe indicated that there were approximately *4.9- and 4.5-fold* increased ROS contents in the diabetic arteriolar and venular walls with respect to the control values, respectively (Table 2). The ROS-sensitive fluorescent probe DHR-123 could specifically detect *in vivo* ROS and was used successfully by several other mesenteric studies [12–14]. In our study, the real time video images of mesenteric arteriole and venule which were superfused by DHR-123 elicited that 12-wk diabetic mesenteric venular wall provoked more ROS-sensitive fluorescent intensity significantly than arteriolar wall (*P* < 0.05). But this difference did not occur in other experimental groups. Besides, this increased ROS contents existed at the same stage where the increased number of leukocyte adherence was encountered. Therefore, it may implicate that the significant* increased number of leukocyte adherence on 12-wk DM venules might be the secondary source* of ROS generation, particular via the NADPH oxidase, and then caused more ROS-associated fluorescent intensity on venular wall than diabetic arteriolar wall.

In addition, when the correlation between ROS contents and vascular function was examined, our findings shown in Figures 4(a) and 4(b) indicated that the relationships of those parameters were significant (*P* < 0.02). The results indicated that: (1) the negative correlation between the ROS generation and vasorelaxation was obtained by the linear line, *y*_*a*_ = −0.101*x*_*a*_ + 16.68 (*R* ^2^ = 0.99, *P* < 0.02); where, (2) the positive correlation between the ROS production and leukocyte adhesion was demonstrated by the linear line, *y*_*v*_ = 0.044*x*_*v*_ + 1.29 (*R* ^2^ = 0.98, *P* < 0.018). The idea was that vascular reactivity has a linear reverse-correlation with arteriolar-wall ROS content. In contrast, leukocyte adherence has a linear correlation with venular-wall ROS content. Several studies [4, 5, 7–9] have indicated that chronic hyperglycemia increased oxidative stress was the major underlining cause which contributed to the impairment of endothelial functions or called endothelial dysfunction (ED). Similar to our study, ED was characterized by the results of endothelium-dependent vascular relaxation and the leukocyte-endothelium interaction (Figures 2-3). Interestingly, our findings have also demonstrated that diabetes-induced ED could be either *prevented* (the results of DM + AA2day) or *improved* (the result of DM + AA6week) by vitamin C supplementation. Therefore, these findings implied that as long as AA could diminish diabetes-induced ROS content, the activity of endothelium was able to be restored, probably via the maintenance of nitric oxide (NO) bioactivity. Endothelial nitric oxide (NO) bioactivity has a crucial role on both vascular sites, since, NO is able to regulate both endothelium dependent vasorelaxation and suppressing adhesive molecules expression [21–25]. The previous report using NO fluorescent indicator, DAF-2DA, has shown this benefit of AA supplementation on increased NO bioavailability as well [26]. However, the exact mechanisms between roles of antioxidant and consequent inflammatory process-derived-ROS production still need to be clarified.

Several studies indicate a close correlation between BH_4_ bioavailability and eNOS function. Particularly, in diabetes the reduction of bioavailability of BH_4_ promoted eNOS uncoupling which contributes to further reduced NO bioavailability and generates O_2_− and/or H_2_O_2_ rather than NO.

In recent study, they found a significant reduction of intracellular BH_4_ levels after challenging endothelial progenitor cells (EPCs) with high glucose concentration, whereas, oxidized BH_2_ levels significantly increased [27]. Consistent to the previous study by Sinozaki et al., 1999, they showed that high fructose-fed rats enhanced formation of O_2_−, which was caused by relative deficiency of BH_4_ and elevation of BH_2_ levels in aortic tissue [28]. Based on this review, it is hypothesized that diabetes-induced ROS could be related to the deficiency of BH_4_ by O_2_− oxidation, resulting in enhanced BH_2_ (BH_4_ + O_2_− → BH_2_ + H_2_O_2_). Therefore, we conducted the further study by using BH_4_ synthetic inhibitor, DAHP, in order to imitate the diabetic rat model. As shown in Table 3, the 30-min DHAP administration, the ROS-dependent fluorescence intensities were significantly increased up to ~3.1 time when compared to CON+veh group. Whereas the combination of BH_4_ and AA (DAHP+BH_4_+ AA- treated CON group) could decrease ROS intensity as much as 80% of CON+DAHP group.

Besides, the percentage changes of arteriolar diameters after Ach application could increase significantly after BH_4_+AA-treated. In addition, our findings shown in Figure 5 indicated that the relationships between ROS contents and vascular function could be represented by a linear equation; *y* = −0.097*x*+23.45 (*R* ^2^ = 0.61). Therefore, it might conclude that vitamin C can improve the uncoupled eNOS and enhance its enzymatic activity as demonstrated by increased Ach-induced vasodilation which was associated with the decreased ROS contents, Since vitamin C could reduce the BH_2_ to regenerate BH_4_ by the following action: HO-ASC-OH + BH_2_ → O = ASC = O + BH_4_ [28–31].

**Figure 6:**
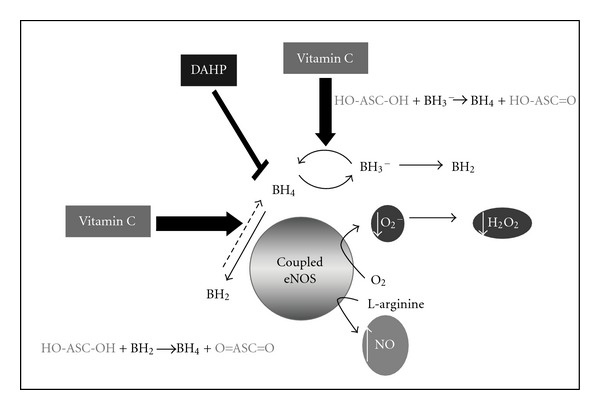
The diagram represents the proposed mechanism of ascorbic acid (AA) in attenuating reactive oxygen species (ROS) production when DAHP inhibited BH_4_ and caused eNOS uncoupling. Similar to diabetic-induced endothelial cells dysfunction in associated with BH_4_ deficiency.

At this point, it may be suggested that in the situation of DAHP-induced BH_4_ deficiency, eNOS uncoupling is produced as it will generate O_2_− and consequently convert to H_2_O_2_− as demonstrated by DHR-123 detection. In addition when eNOS uncoupling, less of NO is produced, therefore, the Ach-induced vasodilatation becomes significantly decreased in CON + DAHP group. In Figure 6, this idea is summarized and presented for supporting the beneficial mechanism of vitamin C on protecting endothelial function against oxidative stress condition.

At present, a number of human researches have focused on preventing diabetes-induced cardiovascular complications using antioxidants such as vitamins B, C, and E. As their known superoxide scavenger properties, vitamins C and E have been widely investigated either alone or in combination. However, the beneficial effects of vitamin E supplementation in surrogate measurements of cardiovascular disease in diabetics have not shown any clear results [32–34]. A study from our unit, which included both types I and II diabetic patients treated with a high dose of vitamin E (1,800 IU daily) for 12 months, found no improvement in endothelial-dependent or-independent vasodilation in both skin microcirculation and brachial artery microcirculation tests. [33] In addition, left ventricular function was not affected by vitamin E supplementation. In other word, vitamin C is also thought to be involved in recycling the *α*-tocopheryl radical back to *α*-tocopherol [35].

In conclusion, our findings have provided the *in vivo *evidences for the fact that the abnormality of endothelial cell observed in DM rats is significantly in relation to the ROS generation in microvascular walls. In particular, our findings have indicated that microvascular ROS content seem to be a major indicator for the progression of diabetes-induced endothelial dysfunction. Therefore, the reduction of microvascular ROS content by antioxidant like vitamin C may be a valuable therapeutic approach in preventing and reversing diabetic-induced microvascular dysfunction.

## Figures and Tables

**Table 1:** Means ± SE of blood glucose (BG), HbA_1C_, body weight (BW), plasma ascorbic acid (Plasma AA), mean arterial blood pressure (MAP) were demonstrated for 12 weeks of experimental periods control, DM, DM + AAday2, DM + AA6wk.

Group	BG (mg/dL)	HbA_1C_ (%)	BW (g)	Plasma AA (mg/dL)	MAP (mmHg)
Control	93.13 ± 7.70 (*n* = 8)	3.68 ± 0.54 (*n* = 6)	425.67 ± 4.46 (*n* = 9)	1.30 ± 0.15 (*n* = 6)	97.22 ± 6.68 (*n* = 8)
DM	418.33 ± 17.24* (*n* = 9)	10.86 ± 0.24* (*n* = 7)	182.70 ± 7.87* (*n* = 10)	0.62 ± 0.02* (*n* = 7)	113.54 ± 3.16* (*n* = 9)
DM + AAday2	380.13 ± 18.61 (*n* = 8)	9.81 ± 0.30 (*n* = 8)	228.00 ± 15.18 (*n* = 9)	0.99 ± 0.03^#^ (*n* = 5)	92.70 ± 6.47^#^ (*n* = 8)
DM + AA6wk	407.80 ± 28.8 (*n* = 5)	10.34 ± 0.35 (*n* = 5)	283.75 ± 22.65 (*n* = 6)	1.27 ± 0.04^#^ (*n* = 5)	95.33 ± 6.90^#^ (*n* = 7)

*P < 0.001, significant difference compared to control.

^#^ *P* < 0.001, significant difference compared to DM.

**Table 2:** Means ± S.E. of changes in DHR fluorescence intensities along the arterioles and venules. Values are means ± SE of each group, CON (*n* = 5), DM (*n* = 5), DM + AAday2 (*n* = 6), and DM + AA6wk (*n* = 5), calculated by using the equation of: % change of ROS-associated fluorescent intensity = (*I*_1 min_ − *I*_base_/*I*_base_) × 100.

Group	% change of ROS in arteriolar wall	% change of ROS in venular wall
Control	34.08 + 15.62	38.31 + 18.71
DM	165.89 + 24.59*	172.26 ± 34.70^∗,a^
DM + AAday2	32.86 + 14.56^#^	49.59 ± 21.26^#^
DM + AA6wk	26.02 + 8.03^#^	49.91 ± 20.10^#^

*P < 0.001, significant difference compared to control.

^#^ *P* < 0.001, significant difference compared to DM.

^a^ *P* < 0.05, significant difference between ROS contents in arteriolar wall and venular wall in DM group.

**Table 3:** Percentage changes of ROS (intensities/100-**μ**m arteriolar length) and arteriolar diameters in response to Ach from mesenteric arterioles in the presence or absence of BH_4_ synthetic inhibitor (DAHP, 20 mM), DAHP plus AA, and DAHP plus BH_4_ donor (0.1 mM) in combination of vitamin C in control (CON) rat group.

Group	% changes of ROS (intensities/100-*μ*m arteriolar length)	% changes of arteriolar diameters
	6-wks CON	
CON + Vehicle	54.48 ± 14.90 (*n* = 4)	13.22 ± 1.24 (*n* = 8)
CON + DAHP	169.61 ± 6.46*** (*n* = 4)	4.34 ± 0.78*** (*n* = 7)
CON + DAHP + AA	115.66 ± 10.96^aaa^ (*n* = 3)	17.98 ± 1.42^aaa^ (*n* = 6)
CON + DAHP + BH_4_ 0.1 mM + Vit. C	33.89 ± 7.62^aaa^ (*n* = 3)	22.10 ± 1.41^aaa^ (*n* = 6)

Values are mean ± SEM.

***P < 0.001, significantly difference compared to 6-wk vehicle.

^a^ *P* < 0.05,  ^aaa^ *P* < 0.001, significantly difference compared to 6-wk DAHP.
